# Recoverability of Diabetic Nephropathy of Donor Kidney After Kidney Transplantation

**DOI:** 10.3389/ti.2022.10714

**Published:** 2022-09-15

**Authors:** Kyo Won Lee, Jongmin Sim, Sean S. W. Park, Junseok Jeon, Gyuri Kim, Min Jung Kim, Ghee Young Kwon, Hye Ryoun Jang, Wooseong Huh, Jae Berm Park

**Affiliations:** ^1^ Department of Surgery, Samsung Medical Center, Sungkyunkwan University, Seoul, South Korea; ^2^ Department of Pathology, Anam Hospital, Korea University, Seoul, South Korea; ^3^ Division of Nephrology, Department of Medicine, Samsung Medical Center, Sungkyunkwan University, Seoul, South Korea; ^4^ Division of Endocrinology and Metabolism, Department of Medicine, Samsung Medical Center, Sungkyunkwan University, Seoul, South Korea; ^5^ Department of Surgery, Seoul Medical Center, Seoul, South Korea; ^6^ Department of Pathology, Samsung Medical Center, Sungkyunkwan University, Seoul, South Korea

**Keywords:** kidney transplantation, biopsy, donor, diabetic nephropathy, glomerulus basement membrane

## Abstract

Some kidney donors have diabetes, and little of their natural course of diabetic nephropathy (DN) is known. The aim of this study was to analyze the changes in pathologic lesions in the diabetic donor kidney after KT by performing protocol biopsy two weeks and one year after KT. This retrospective study included 103 patients who underwent KT, with kidneys from donors with a history of diabetes mellitus (DM). Among them, data of 34 patients who underwent biopsy two weeks and one year after KT were reviewed. Biopsy specimens were reviewed using light microscopy and electron microscopy. Glomerular basement membrane (GBM) thickness at 2 weeks and 1 year was compared. Biopsy showed that DN occurred in 29 of the 34 patients. Only trivial histological changes were observed in 22 patients (64.7%), including 5 patients who did not show DN. At one year after transplantation, there was no change in the DN histologic class in 26 patients (76.5%), and there was no statistically significant difference in the change in GBM thickness. This pattern was observed regardless of the recipient’s DM or glycemic control. With this understanding, clinicians can use kidneys from DM donors with more comfort, thereby reducing the kidney discard rate.

## Graphical Abstract

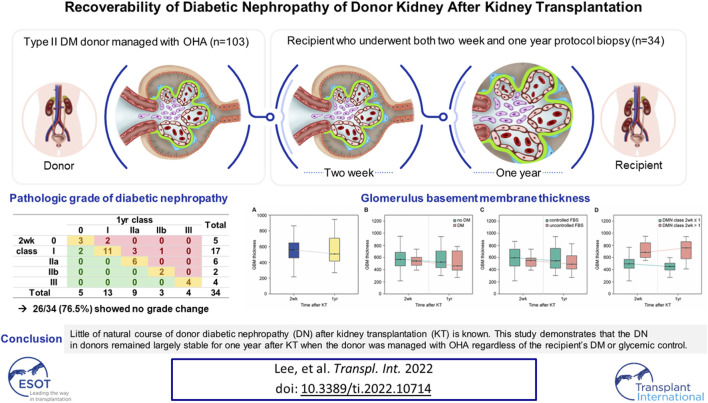

## Introduction

Several studies have demonstrated that kidney transplantation is the treatment of choice in patients with end-stage renal disease. Therefore, attempts have been made to implement more kidney transplantations (KTs) and expand the donor criteria. With the expansion of the donor criteria, the number of KT with a diabetic donor kidney is also increasing. The effect of diabetic donors on the outcome of KT is controversial. A study by Mohan et al showed that diabetes mellitus (DM) alone in donors did not appear to have any effect on death-censored graft survival (1). but in a study by Ahmad et al, there was a significant difference in death-censored graft survival depending on the presence or absence of DM in the donor. Although statistically significant, the 10-year death-censored graft survival was not considerably different, with 57.1% in the DM group and 54.6% in the non-DM group (2). By analyzing the United Network for Organ Sharing (UNOS) registry data, Cohen et al confirmed that allograft survival was significantly lower when a kidney from a diabetic donor was used, and reported that the difference in allograft survival was also significantly affected by the presence or absence of DM in the recipient (3). These results suggest that diabetic nephropathy (DN) is affected by glycemic control.

DN in patients with type 1 DM may be reversible when diabetes is cured by pancreas transplantation (4). However, it is not well documented whether pathologic changes in DN in type 2 DM can also be reversible, as in type 1 DM. It is difficult to evaluate the reversibility of pathologic lesions of DN in patients with type 2 DM because there is no established treatment that cures type 2 DM, and these patients often have several comorbidities that can affect kidney disease, including metabolic syndrome.

In recent years, many efforts have been made to reduce the donor kidney discard rate, and understanding the natural course of donor DN is essential to reduce the discard rate in the reality that more than 40% of kidneys from diabetic donors are discarded (5). However, only few studies have evaluated the pathologic status and changes in the kidneys of DM donors and these studies included a small number of patients with DN (6,7,8,9). Therefore, to reach a more robust conclusion, biopsy results at regular intervals are needed in a larger number of patients.

The aim of this study was to analyze the changes in pathologic lesions in the DM donor kidney after KT in a large number of patients and for the same 1-year duration by performing biopsy at 2 weeks and 1 year after KT. In addition, the difference in the change according to the recipient’s DM status, glycemic control, and severity of donor kidney DN was also determined.

## Materials and Methods

### Study Design

Among the patients who underwent KT between January 2013 and December 2018 at Samsung Medical Center, 103 recipients received kidneys from donors with a history of DM but only 37 recipients completed full sets of post-transplant protocol biopsies. A retrospective review of those patients was carried out, and three patients were excluded as their graft tissue samples were inappropriate for assessment, leaving 34 patients for the final analysis. In our center, we perform post-transplant protocol biopsy at 2 weeks and 1 year. The biopsy tissue at 2 weeks is considered to reflect the donor’s DN status, and the tissue at 1 year the recipient’s glycemic control status over the first year after KT. The protocol biopsy is contraindicated if the patient does not consent or if percutaneous coronary intervention had been performed within the preceding year of surgery, requiring ongoing anticoagulation. Pediatric cases and donation after circulatory death were excluded from the study as they are not routinely included in our protocol biopsy.

Recipient DM was defined as a history of DM or DM medication requirement after transplantation. Uncontrolled fasting blood sugar (FBS) was defined as an FBS level of ≥126 mg/dl, which was observed two times or more from 2 months after KT when the maintenance steroid dose (methylprednisolone 4mg per day) was being administered.

The institutional review board of Samsung Medical Center approved this study protocol (SMC 2020–12-139) and waived the requirement for obtaining patients’ written informed consent because of the retrospective nature of the study and as the data used were anonymized.

### Post-Transplant Management

For induction immunosuppression, basiliximab (20 mg/day, 2 days) and rabbit antithymocyte globulin (1.5 mg/kg, 3 days) were used. For maintenance immunosuppression, all patients were treated with a triple immunosuppressive regimen of tacrolimus, mycophenolate mofetil, and methylprednisolone. For therapeutic monitoring of tacrolimus, the tacrolimus trough level was monitored and the dosage was adjusted to maintain a target concentration of 8–10 ng/ml during 1 month post-KT, 5–8 ng/ml during 1 month to 1 year, and 3–7 ng/ml afterward. Methylprednisolone was started on the day of surgery at an intravenous dose of 500 mg/day and administered for 2 days and then tapered by half every day to 60 mg/day. Oral methylprednisolone was administered at 32 mg/day for 7 days, 16 mg/day for the next 2 weeks, 8 mg/day for the next month, and 4 mg/day for maintenance. Post-transplant steroids were gradually tapered off and totally withdrawn 6 months after KT.

Blood glucose measurements were continued using a glucometer 4 times a day in patients who underwent kidney transplantation. In most cases, after administration of high-dose steroids, there was a rapid rise in blood glucose level, and when pre-meal blood glucose levels continued to exceed 200 mg/dl, multiple daily insulin injections were started and the insulin dose was titrated according to the blood glucose level. If the pre-meal blood glucose level was 150–200 mg/dl, oral hypoglycemic agents were used. Subsequent reductions in the steroid dose according to the immunosuppressive protocol resulted in a decrease in insulin requirements and a 10%–20% reduced insulin dose was administered. When the low-dose steroid was maintained and the blood glucose was well controlled, insulin administration was switched to oral hypoglycemic agents or discontinued.

### Histologic Assessment of DN

The assessment of renal biopsy specimens was undertaken by a specialist renal pathologist, who was blinded to the clinical details. Biopsy specimens were reviewed by light microscopy (LM) and electron microscopy (EM). Immunofluorescence staining results were reviewed with the pathology reports. Hematoxylin and eosin staining, and periodic acid-Schiff staining were performed for LM sections. The details of the histopathological features examined are as follows: the number of total glomeruli/globally and segmentally sclerotic glomeruli, as well as mesangial expansion. The histological examinations were performed twice under LM before reaching the final classification of DN. During the process, we encountered a discrepancy in only one case, where an additional independent assessment was performed to resolve the inconsistency. The thickness of the glomerular basement membrane (GBM) was measured through EM, and samples with a relatively uniform thickness were measured at five locations, and non-uniform cases were measured at up to 21 locations; the average of the measured values was used for analysis.

**FIGURE 1:**
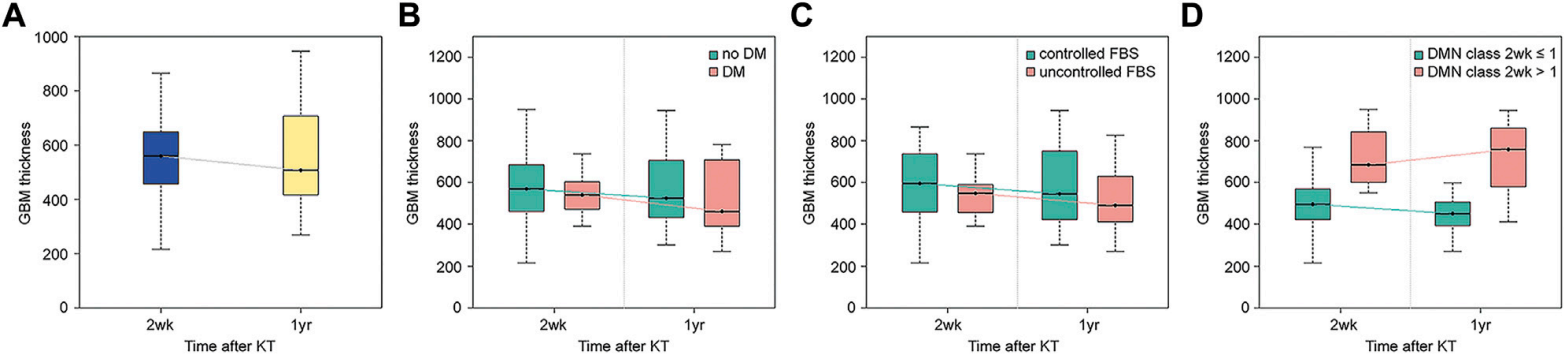
Representative light microscopic images of renal biopsy specimens obtained from patients with kidney transplantation (periodic acid-Schiff stain, ×400). **(A)** Nonspecific change, diabetic nephropathy (DN) class I. Minimal mesangial change was seen. **(B)** Mild mesangial expansion and hypercellularity, DN class IIa. **(C)** Marked mesangial expansion and hypercellularity without nodule formation, DN class IIb. **(D)** Marked mesangial expansion and nodule formation, DN class III.

The criteria suggested in previous studies were used for histological classification of DN (10, 11). Class I was defined as a change in the LM that was insignificant and when the GBM was thickened upon observation under EM (by definition, exceeding the average thickness of 430 nm in males and 395 nm in females). Class II was defined as mesangial expansion seen in >25% of the glomeruli upon observation with LM (mild mesangial expansion was referred to as IIa and severe mesangial expansion was referred to as IIb). Class III was defined as the mesangial expansion to form a nodule, and class IV was defined as global sclerosis in more than half of the glomeruli (Figure 1).

### Statistical Analysis

Statistical analysis was performed using SAS version 9.4 (SAS Institute, Cary, NC, United States) and R 4.0.3 (Vienna, Austria; http://www.R-project.org/) software. GBM thickness at 2 weeks and that at 1 year were compared using paired t-test and Wilcoxon signed-rank test, and logistic regression test was used for the DN progression risk-factor analysis. Cox regression test was used for the graft failure risk-factor analysis. Statistical significance was defined as a *P*-value < 0.05.

## Results

The donor and recipient information of the 34 cases is summarized in Table 1. The mean age of donors was 60.4 years, 94% (32/34) were brain-dead donors, and the pre-transplant serum creatinine level was 1.6 ± 0.9 mg/dl. All donors had insulin independent type 2 DM, 31 (91.2%) of whom were on oral hypoglycemic agent (OHA). The mean age of recipients was 53.8 years, and 32.4% (11/34) had a history of DM.

**TABLE 1:** Patient characteristics.

	*N*
Donor age (years, mean ± SD)	60.38 ± 9.53
Male donor (n, %)	24 (70.6)
Donor BMI (kg/m^2^, mean ± SD)	24.27 ± 3.7
Donor HTN (n, %)	20 (58.8)
Terminal creatinine (mg/dL, mean ± SD)	1.6 ± 0.9
Donor DM duration (years, median [range])	7.5 [1.0, 22.0]
Donor DM medication	
OHA (n, %)	31 (91.2)
No treatment (n, %)	2 (5.9)
Unknown (n, %)	1 (2.9)
Donor HbA1c (%, mean ± SD)	6.9 ± 1.4
Donor proteinuria^a^ (n, %)	13 (30.2)
LD/DD	2/32
Recipient age (years, mean ± SD)	53.82 ± 10.68
Male recipient (n, %)	20 (58.8)
Recipient BMI (kg/m^2^, mean ± SD)	22.87 ± 2.97
Recipient diabetes (n, %)	11 (32.4)
Recipient HTN (n, %)	29 (85.3)
Cause of ESRD (n, %)	
DM	10 (29.4)
HTN	3 (8.8)
GN	4 (11.8)
Others	17 (50.0)
Patients with previous transplants (n, %)	3 (8.8)
dialysis duration (day, mean ± SD)	2337.09 ± 1032.23

SD, standard deviation; BMI, body mass index; HTN, hypertension; LD, living donor; DD, deceased donor; ESRD, end-stage renal disease; GN, glomerulonephritis.

a Donor proteinuria was defined when dipstick ≥2+.

One patient was found to have stage I membranous glomerulonephritis (MGN), with concurrent DN (12). This patient was diagnosed with Class IIa DN on both protocol biopsies at 2 weeks and 1 year. The rest of the patients were negative for immunoglobulin G (IgG), IgA, IgM, complement 1q (C1q), C3, and C4. Furthermore, electron dense deposit was not present in all other patients except for the one with MGN.

### Changes in the DN Histologic Class

Table 2 summarizes the changes in the DN histologic class in the biopsy at 2 weeks and 1 year. At the 2-week biopsy, five patients were classified as having class 0 (no DN), 17 as having class I, 6 as having class IIa, two as having class IIb, 4 as having class III, and none as having class IV. None of the donor characteristics including age, duration of DM, HbA1c were not associated with the DN class (Supplementary Table S1). The donors whose DN class was III were found to have had DM for more than 6 years (Supplementary Figure S1). At the 1-year biopsy, 5 patients were classified as having class 0, 13 as having class I, 9 as having class IIa, 3 as having class IIb, 4 as having class III, and none as having class IV.

**TABLE 2:** Change in diabetic nephropathy histologic class.

		1-Year class					Total
		0	I	IIa	IIb	III	
**2-Week class**	**0**	3	2	0	0	0	5
	**I**	2	11	3	1	0	17
	**IIa**	0	0	6	0	0	6
	**IIb**	0	0	0	2	0	2
	**III**	0	0	0	0	4	4
**Total**		5	13	9	3	4	34

Between 2 weeks and 1 year, the histologic class regressed in two patients (6.9%) and progressed in 6 patients (17.6%), and there was no change in the histologic class in 26 patients (76.5%). In most cases, there was no change in histologic class, and this pattern did not change even when classifying recipients according to DM or FBS control status. There was no change in histologic class in 9 out of 11 recipients with DM (81.8%), and no change in histologic class in 17 out of 23 recipients without DM (73.9%). The number of patients who showed regression was one in each group, and the number of patients who showed progression was 5 (21.7%) in the non-DM group and one (9.1%) in the DM group (Table 3). The same pattern was observed when the patients were classified according to their FBS control status. Class change was not observed in 13 (81.3%) of the 16 patients with controlled FBS, and there was no change in class in 13 (72.2%) of the 18 patients with uncontrolled FBS. Regression occurred in only two patients with uncontrolled FBS, and progression occurred in three patients in each group (controlled FBS, 23.1% and uncontrolled FBS, 16.7%) (Table 4). The status of recipient DM or uncontrolled FBS was not evaluated as a risk factor of the histologic grade progression of DN (Supplementary Table S2).

**TABLE 3:** Change in diabetic nephropathy histologic class according to recipient DM status.

		Non-DM recipient						DM recipient					
		1-Year class					Total	1-Year class					Total
		0	I	IIa	IIb	III		0	I	IIa	IIb	III	
**2-Week class**	**0**	2	2	0	0	0	4	1	0	0	0	0	1
	**I**	1	6	2	1	0	10	1	5	1	0	0	7
	**IIa**	0	0	5	0	0	5	0	0	1	0	0	1
	**IIb**	0	0	0	2	0	2	0	0	0	0	0	0
	**III**	0	0	0	0	2	2	0	0	0	0	2	2
**Total**		3	8	7	3	2	23	2	5	2	0	2	11

DM, diabetes mellitus.

**TABLE 4:** Change in diabetic nephropathy histologic class according to recipient FBS control status.

		Controlled FBS						Uncontrolled FBS					
		1-Year class					Total	1-Year class					Total
		0	I	IIa	IIb	III		0	I	IIa	IIb	III	
**2-Week class**	**0**	2	2	0	0	0	4	1	0	0	0	0	1
	**I**	0	3	0	1	0	4	2	8	3	0	0	13
	**IIa**	0	0	5	0	0	5	0	0	1	0	0	1
	**IIb**	0	0	0	2	0	2	0	0	0	0	0	0
	**III**	0	0	0	0	1	1	0	0	0	0	3	3
**Total**		2	5	5	3	1	16	3	8	4	0	3	18

FBS, fasting blood sugar.

### Change in GBM Thickness

**FIGURE 2:**
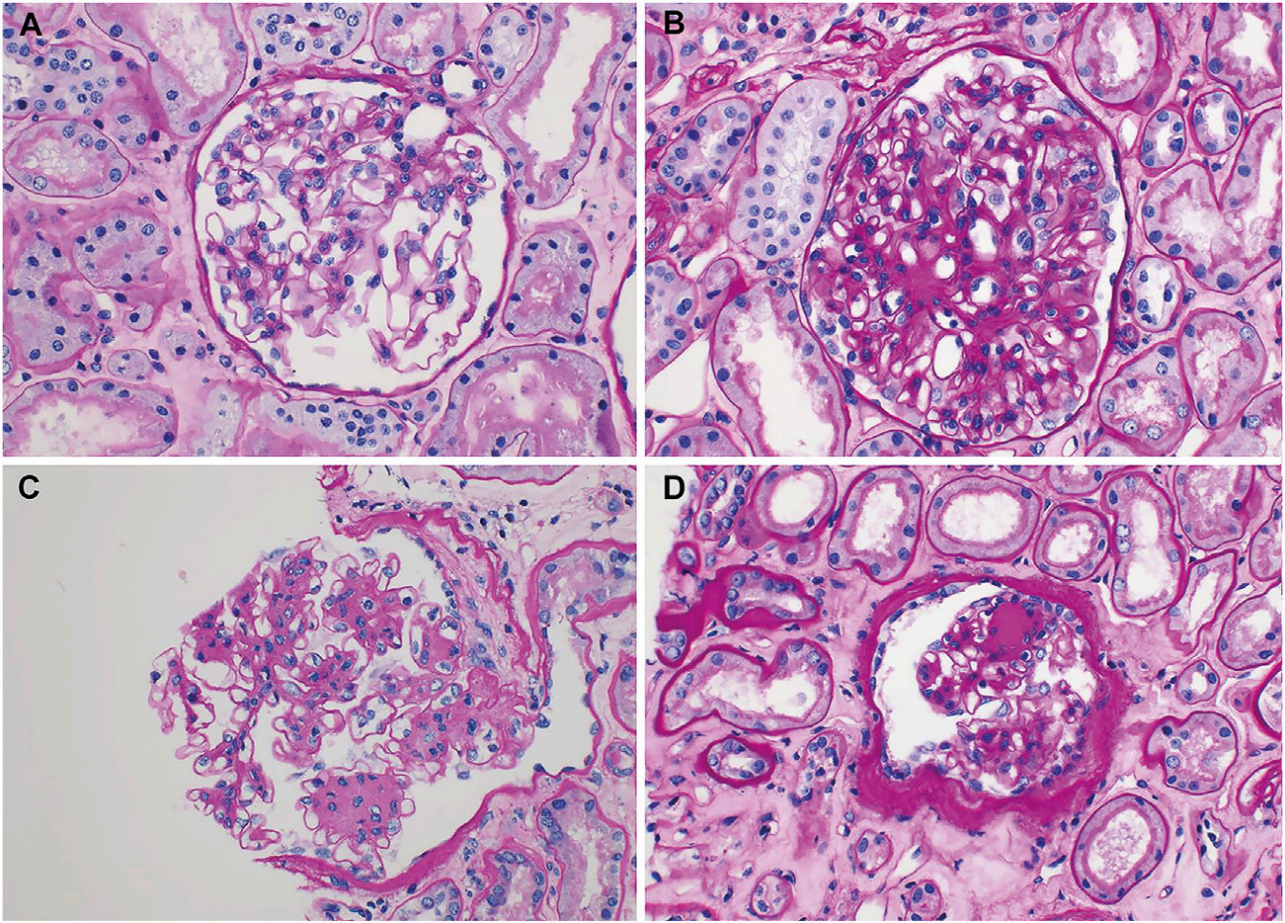
Representative electron microscopic images of renal biopsy specimens obtained from kidney transplantation patients with nonspecific changes (diabetic nephropathy, grade 0 or I) in the light microscope and a change in the electron microscope. In addition to the typical measurement values shown in the images, the average value was obtained by additionally measuring for up to 16 points. **(A,B)** Progression; **(A)** segmental and mild thickening of the glomerular basement membrane (GBM), measuring 318–511 nm (375 nm in mean) (original magnification, ×4000) and **(B)** uniformly thickened GBM, measuring 395–611 nm (536 nm in mean) (original magnification, ×3500). **(C,D)** Regression; **(C)** marked thickening with segmental normal thickness of the GBM, measuring 254–767 nm (600 nm in mean) (original magnification, ×5000) and **(D)** marked, but segmental thickening of the GBM, measuring 208–562 nm (372 nm in mean) (original magnification, ×6000).

**FIGURE 3:**
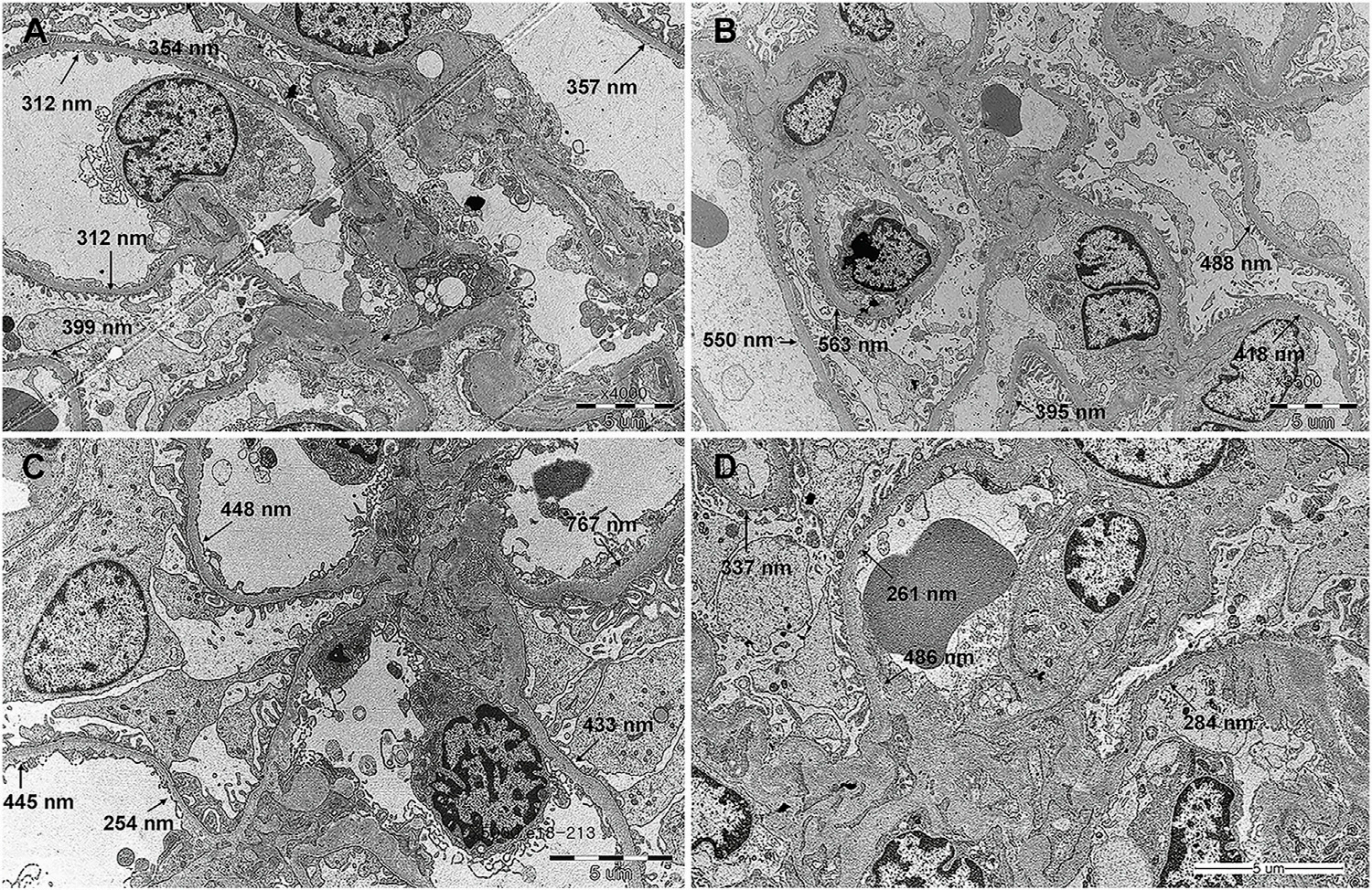
Change in glomerulus basement membrane thickness from 2 weeks to 1 year after kidney transplantation. **(A)** All patients. **(B)** Patients were divided according to recipient diabetes status. **(C)** Patients were divided according to fasting blood sugar control status. **(D)** Patients were divided according to diabetic nephropathy histologic class at 2 weeks after kidney transplantation.

Compared with the GBM thickness measured at the 2-week biopsy, that at 1-year biopsy decreased in 21 patients (Figures 2A,B) and increased in 13 patients (Figures 2C,D). The mean GBM thickness showed a decreasing trend, but the difference was not statistically significant (Figure 3A, *p* = 0.29). When patients were classified based on the presence or absence of recipient DM, the thickness increased in 3 out of 11 patients (27.3%) with DM and decreased in 8 (72.7%). The average thickness at both time points decreased in both groups, but the difference was not statistically significant (Figure 3B, *p* = 0.21 and 0.73, respectively).

When the patients were classified based on recipient FBS control status, the thickness increased in 5 out of 18 (27.8%) patients with uncontrolled FBS and decreased in 13 (72.2%), and it increased in 8 out of 16 (50%) patients with controlled FBS and decreased in 8 (50%). The average thickness at both time points appeared to decrease in both groups, but the difference was not statistically significant (Figure 3C, *p* = 0.24 and 0.85, respectively). When the patients were classified based on the severity of DN, the thickness increased in 8 of the 22 patients with class I or lower disease and decreased in 14. Thickness increased in five of the 12 patients with class II or higher disease and decreased in 7. The average thickness decreased in patients with class I or lower disease during both time points and increased in patients with class II or higher disease, but the difference between the mean values was not statistically significant (Figure 3D, *p* = 0.10 and 0.81, respectively).

### Clinical Outcomes After Kidney Transplantation

The median follow-up of the patients was 48.5 months. No patient died during this period, but 6 patients (17.6%) lost their grafts. The mean GFRs at 2 weeks and 1 year were 49.1 ± 22.5, and 51.2 ± 15.3 (mL/min/1.73 m^2^), respectively (Supplementary Table S3). The cause of graft failure was attributed to recurrent rejection and septic shock in two cases, cardiovascular shock after aortic dissection in one case, DN progression in one case. Two cases did not have any obvious cause identified. Among the 6 patients who lost their graft, three patients showed class IV DN at 2-week protocol biopsy and the other three class I DN. A risk factor analysis for graft failure demonstrated that an evidence of class III DN at 2-week biopsy was the only independent risk factor (*p* < 0.001) even though DN was a cause of graft failure in only one patient. However, progression of DN was not a significant risk factor for graft failure with *p* value of 0.61 (Supplementary Table S4).

## Discussion

We have analyzed how donor DN changes over the year after KT. Pathological biopsy of patients who received KT from 34 DM donors showed that DN occurred in 29 of the 34 patients. However, 17 of them (50% of the total patients) were classified as having class I, a mild case with only an increase in GBM thickness observed under EM. Minor histological changes were observed in 22 patients (64.7% of the total), including 5 patients who did not show DN. At 1 year after transplantation, there was no change in the DN histologic class in 26 patients (76.5%), and there was no statistically significant difference in the change in GBM thickness. This pattern was observed regardless of the recipient’s DM or FBS control status.

Based on a study by Fioretto et al that reported improved DN after pancreas transplantation, DN is known to improve with good glycemic control (4, 13, 14). In this study, the histological findings of DN improved when the blood sugar levels were normalized by pancreas transplantation; this was not a short-term phenomenon, and histological improvements occurred 10 years after the transplantation. Abouna et al reported a case in which histological improvements occurred following KT with a donor’s kidney with DN in a non-DM patient (15). Similarly, Harada et al investigated how histological lesions changed over a year after good glycemic control in three non-diabetic recipients who underwent KT with donor kidneys showing early diabetic nephropathy (two class I patients and one class IIa patient). The recipients who had pre-existing DM, or who developed post-transplant DM (PTDM) or new-onset diabetes after transplantation (NODAT), were excluded. The study demonstrated that the early diabetic changes in the graft improved in all patients after good glycemic control post KT. However, in this study, even in patients with no history of DM or PTDM (*n* = 23), the class of DN was stable or progressed after 1 year of KT (Table 3), and the change in GBM thickness was also not significant (Figure 3B). The class of DN was found to remain stable or progress (Table 4) even in the group that was selected more stringently, which excluded those with uncontrolled FBS (*n* = 16), and the change in GBM thickness was not significant (Figure 3C). This may be because the period of 1 year was short, as changes in glycemic control for a sufficient period are required to induce histologic changes in DN, as stated by Fioretto et al.

The incidence of PTDM is quite high owing to the use of immunosuppressants after KT (16, 17). In a multicenter study, Porrini et al conducted an oral glucose tolerance test every year in 672 patients for up to 5 years after KT and confirmed that PTDM occurred in 32% of the patients, and in nearly half of the patients when prediabetes was included (16). Therefore, it is difficult to generalize the results of Harada et al in the field of KT. Truong et al confirmed that DN was stable or progressed slowly through post-perfusion and follow-up biopsies. Three patients were confirmed to be stable, and four patients who were confirmed to have disease progression had PTDM (8). By analyzing the UNOS registry data, Cohen et al confirmed that allograft survival was significantly lower when a kidney of a diabetic donor was used, and reported that the difference in allograft survival was also significantly affected by recipient DM (3). These results suggest that DN is affected by glycemic control. However, the results of this study showed that the changes in the histologic class of DN after 1 year of KT did not differ depending on the status of DM or FBS control (Tables 3, 4), and changes in GBM thickness did not show any different patterns depending on the status of DM or FBS control (Figures 3B,C). This could be simply due to the previously mentioned insufficient duration of follow up. But It is also possible that the poor outcome of the recipient with DM when diabetic donor kidney was used is caused by reasons other than the histological evidence of deterioration alone. Therefore, long-term data on the natural course of donor DN are required to verify this.

Hsu et al reported that donor DN is transmissible to recipients (9). DN was transmitted in five of the six cases with donor DN, and the histologic class of DN progressed in three of the five cases. The recipient in whom DN was not transmitted had no DM history, no PTDM, and a level of HbA1c maintained below 6% after transplantation. In the 5 cases in which DN transmission occurred, the recipients had a high histologic class of DN (one class IIa patient, two class IIb patients, and two class III patients). In this study, the changing pattern of GBM thickness also showed different patterns depending on the histologic findings at 2 weeks after KT. If the tissue class was I or lower at 2 weeks, the average thickness decreased, similar to the overall pattern, but if the class was II or higher, the average thickness showed an increasing pattern (Figure 3D). Although only one out of three graft failures in the study was directly caused by DN, while the other two by recurrent rejection complicated by sepsis and cardiovascular shock after aortic dissection, the risk of graft failure was higher if the DN class was III at 2 weeks (Supplementary Table S4). And the DM donors with DN of class III had DM duration of at least 6 years (Supplementary Figure S1). This suggests that identifying the class of DN in DM donors through donor kidney biopsy can potentially help predict the prognosis of non-diabetic recipients with DM donor kidneys, especially when the duration of DM was longer than 6 years. This should be confirmed through further research as statistical significance was not demonstrated in this study.

This study has a few limitations. First, given that we only used data from a single institution, the sample size was small. Second, there could be a selection bias as the donors with severe DN would have been clinically unsuitable for KT, and consequently excluded from the study. Hence, the findings from this study are primarily applicable to the insulin independent diabetic donors who are on OHA treatment. Third, we followed up the histological changes for 1 year only. Finally, the possibility of combined idiopathic nodular glomerulosclerosis secondary to smoking, obesity, or other reasons, cannot be completely excluded (18). However, in the current state, where less is known about the course of donor DN after KT, this study will provide important clues in understanding the natural course of donor DN as it monitored the changes during the same period of 1 year using the highest number of tissue findings of DN reported till date. More long-term data of histological changes are needed to improve our understanding of the natural course of donor DN after KT.

## Conclusion

This study demonstrates that the DN in donors remained largely stable for 1 year after KT when the donor with the type 2 DM donor was only managed with OHA. This finding was true, regardless of the recipient’s DM status or how well FBS control was achieved. With this understanding, clinicians may feel more comfortable accepting kidneys from donors with diabetes mellitus, thereby reducing the kidney discard rate. However, long-term follow up data are warranted to better understand the natural course of DN present in donors.

## Data Availability

The original contributions presented in the study are included in the article/Supplementary Material, further inquiries can be directed to the corresponding author.

## Abbreviations

EM, electron microscopy; DM, diabetes mellitus; DN, diabetic nephropathy; FBS, fasting blood sugar; GBM, glomerular basement membrane; KT, kidney transplantation; LM, light microscopy; MGN, membranous glomerulonephritis; PTDM, post-transplant diabetes mellitus.
