# Supplementary material for: Recoverability of Diabetic Nephropathy of Donor Kidney After Kidney Transplantation
Source: Transpl Int. 2022 Sep 15;35:10714. doi: 10.3389/ti.2022.10714 (PMC9519853; doi:10.3389/ti.2022.10714)

**Supplementary Table S1 |** Correlation between donor characteristics and severity of DN at 2 week protocol biopsy.

|  | **Class I**  **(N = 22)** | **Class IIa**  **(N = 6)** | **Class IIb**  **(N = 2)** | **Class III**  **(N = 4)** | ***p*-value** |
| --- | --- | --- | --- | --- | --- |
| DM duration  (year, median [range]) | 6.5 [1.0, 22.0] | 7.5 [3.0, 20.0] | 8.0 [8.0, 8.0] | 8.0 [6.0, 10.0] | 0.92 |
| DM medication (%) | 19 ( 90.5) | 6 (100.0) | 2 (100.0) | 4 (100.0) | 1.00 |
| HgA1C (%, median [range]) | 6.3 [5.1, 8.4] | 6.9 [6.5, 8.9] | 7.2 [6.6, 7.7] | 7.8 [5.8, 12.4] | 0.106 |
| Donor age (year, mean ± SD) | 62.2 ± 8.7 | 56.2 ± 13.6 | 59.0 ± 12.7 | 57.0 ± 5.9 | 0.592 |

*DM, diabetes mellitus; DN, diabetic nephropathy.*

**Supplementary Table S2 |** Diabetic nephropathy progression risk-factor analysis.

|  | ***P*-value** | **Odds ratio** | **95% CI** | |
| --- | --- | --- | --- | --- |
| Donor age | 0.71 | 1.02 | 0.93 | 1.12 |
| Donor BMI | 0.78 | 0.97 | 0.76 | 1.24 |
| Donor HTN | 0.67 | 1.5 | 0.24 | 9.59 |
| Donor DM duration | 0.40 | 1.07 | 0.91 | 1.25 |
| Male donor | 0.46 | 2.37 | 0.24 | 23.36 |
| Recipient age | 0.36 | 0.96 | 0.89 | 1.04 |
| Recipient BMI | 0.41 | 1.15 | 0.82 | 1.60 |
| Recipient DM | 0.38 | 0.36 | 0.04 | 3.53 |
| Recipient HTN | 0.18 | 0.24 | 0.03 | 1.92 |
| Male recipient | 0.21 | 4.33 | 0.45 | 42.02 |
| Uncontrolled FBS | 0.46 | 2.0 | 0.31 | 12.75 |

*DM, diabetes mellitus; FBS, fasting blood sugar; BMI, body mass index; HTN, hypertension; CI, confidence interval.*

**Supplementary Table S3 |** Clinical outcomes after kidney transplantation using a kidney from DM donors.

|  | ***N* = 34** |
| --- | --- |
| Patients death (n, %) | 0 |
| Graft failure (n, %) | 6 (17.6%) |
| 2wk eGFR (mL/min/1.73m^2^, mean±SD) | 49.1 ± 22.5 |
| 1yr eGFR | 51.2 ± 15.3 |
| 2yr eGFR | 52.3 ± 15.5 |
| 3yr eGFR | 51.4 ± 17.4 |
| 5yr eGRF | 52.2 ± 15.3 |
| F/u duration (month, median [range]) | 48.5 [14.0, 103.0] |

**Supplementary Table S4 |** Graft failure risk-factor analysis.

|  | **Hazard ratio (95% CI)** | ***p*-value** |
| --- | --- | --- |
| Donor sex | 0.62 (0.1,3.71) | 0.598 |
| KDPI | 1.02 (0.96,1.08) | 0.598 |
| Donor DM duration | 1.06 (0.93,1.22) | 0.374 |
| DM nephropathy class III at 2wk | 22.24 (3.65,135.46) | < 0.001 |
| DM nephropathy progression | 0.56 (0.06,5.13) | 0.611 |
| Recipient age | 1.06 (0.96,1.17) | 0.271 |
| Recipient DM | 3.33 (0.56,19.94) | 0.188 |
| Recipient uncontrolled BST | 4.11 (0.46,36.79) | 0.207 |

**Supplementary Figure S1 |** Correlation between donor DM duration and severity of DN at 2 week protocol biopsy


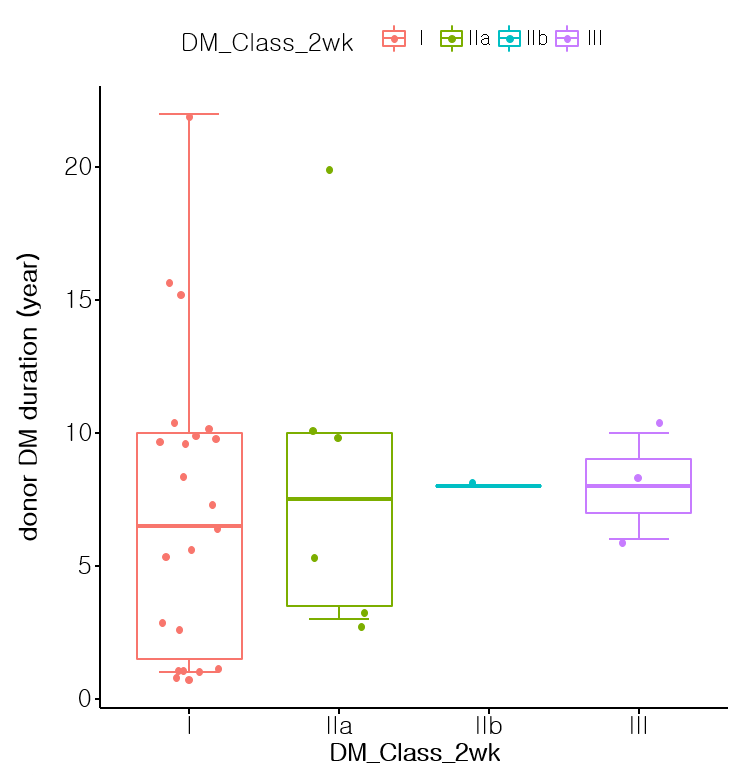

Supplement: Supplementary file 1 [file DataSheet1.docx]
